# Common Neural Substrates for Ordinal Representation in Short-Term Memory, Numerical and Alphabetical Cognition

**DOI:** 10.1371/journal.pone.0092049

**Published:** 2014-03-14

**Authors:** Lucie Attout, Wim Fias, Eric Salmon, Steve Majerus

**Affiliations:** 1 Department of Psychology - Cognition & Behaviour, Université de Liège, Liège, Belgium; 2 Department of Experimental Psychology, Universiteit Gent, Ghent, Belgium; 3 Cyclotron Research Center, Université de Liège, Liège, Belgium; The University of Western Ontario, Canada

## Abstract

The representation and maintenance of serial order information is one of the main functions of verbal short-term memory (STM) but its neural correlates remain poorly understood. We show here that the neural substrates allowing for coding of order information in STM are shared with those supporting ordinal processing in the numerical and alphabetical domains. We designed an fMRI experiment determining the neural substrates sensitive to ordinal distance effects in numerical judgment, alphabetical judgment and serial order STM tasks. Null conjunction analyses for parametric ordinal distance effects showed a common involvement of the horizontal segment of the left intraparietal sulcus over the three tasks; in addition, right intraparietal sulcus involvement was also observed for ordinal distance effects in the STM and numerical judgment tasks. These findings demonstrate that shared neural correlates in the intraparietal cortex support processing of order information in verbal STM, number and alphabetical domains, and suggest the existence of domain general, potentially ordinal, comparison processes supported by the left intraparietal sulcus.

## Introduction

The retention of serial order information, that is, the sequential order in which events have occurred, is a critical dimension of short-term memory (STM), and especially of verbal STM. Recent models of STM but also behavioral, neuropsychological and neuroimaging data highlight the specificity of serial order coding in STM, as opposed to coding of item information (i.e., the phonological and semantic characteristics of the memoranda) [1], [2]. Despite an important number of empirical and modeling studies [3]–[8], the processes supporting serial order coding remain poorly understood. In this study we explore the hypothesis that serial order coding relies on domain general ordinal processes, shared by STM, numerical and non-numerical (alphabetical) domains, and supported by the anterior part of the horizontal segment of the intraparietal sulcus (IPS).

In the STM domain, the anterior part of the horizontal segment of the IPS has been consistently associated with STM for serial order information. Two seminal studies compared item and order recognition for consonant lists and showed that order recognition, as opposed to item recognition, recruited to a larger extent the bilateral IPS as well as premotor frontal areas [9]. More recently, Majerus et al. [5] also compared STM for item and order information with tasks more closely matched with regard to task difficulty and stimulus complexity. They also observed IPS activation for order versus item encoding and retrieval, but with a more specific involvement of the right IPS, the left IPS being equally active during item and order encoding and recognition [4], [10]. The precise function of IPS involvement during serial order coding is, however, currently poorly understood. Many theoretical and computational models of serial order coding have been proposed, with sometimes strongly diverging assumptions. For example, Burgess and Hitch [11], [12] consider that serial order information is encoded via dynamic context signals based on successive list items becoming associated to successive states of a list context signal while Page and Norris [13] consider that serial order coding is related to encoding strength with initial items receiving stronger activation than subsequent items following a primacy gradient; still other models consider that serial order coding is achieved via an oscillator-based timing signal where successive items in a sequence become associated with a network whose activation patterns follow a time-based oscillator [14], or via two dimensional codes, one dimension coding for the start of the list and the other coding for the end of the list. A common denominator of all these models is however the basic assumption of one or several ordinal dimensions as supporting serial order coding. For example, in Brown et al.’s oscillator model, each serial position is associated with a different configuration of the oscillator which evolves following a time-based ordinal progression. The start-end model (SEM) by Henson [7] also uses ordinal coding mechanisms, by associating each item to two ordinal dimensions: one positioning items relative to start of the list, and one positioning items relative to the end of the list; during serial order encoding, each item will be associated with different strength to each of the two dimensions, the first item of the list being strongly associated with the start dimension and weakly with the end dimension, the last item being strongly associated with the end dimension and weakly with the start dimension, and all other items having intermediate weightings relative to these two dimensions, depending on their ordinal position in the memory list. Of central interest for this study, Botvinick and Watanabe [15] proposed a neurocomputational model of STM explicitly assuming that serial order information is coded simply on the basis of ordinal rank information for each item in the memory list. This model considers ordinal coding as being a fundamental property of STM processing with ordinal coding in this model being related to the IPS as the representational hub of ordinal codes, in line with the core hypothesis of the present study [15], [16]. In sum, the vast majority of current STM models for serial order agree on ordinal coding as underlying representation of serial order information, even if the implementation of these ordinal codes varies greatly among the different models.

Numerical cognition is another domain where ordinal processing is a fundamental dimension, each number having a fixed position relative to the other numbers of the mental number line, like the serial positions of items in a STM list. Importantly, IPS involvement is a central neural signature of number processing. The parietal cortex and particularly the horizontal segment of the IPS support number processing [17]–[20]. Moreover, this region is sensitive to numerical distance effects, with smaller activation for far distances and larger activation for close distances, and this both during magnitude (is 7 larger than 5?) and ordinal (does 7 come before 9?) processing of numbers [21], [22]. Interestingly, a similar effect also characterizes STM: response times are faster and accuracy is higher when judging the serial order of two items coming from more distant positions of the STM list as compared to closer positions [9], and this STM distance effect is also associated with activation in left IPS [23]. More generally, other types of information contain an inherently ordinal structure such as the days of the week, the months of the year or the letters of the alphabet. In tasks requiring the judgment of ordinal distances between items of the aforementioned categories, the horizontal segment of the IPS is again activated [24]–[26]. In sum, these different studies indicate that the horizontal segment of the IPS may support domain general ordinal processing shared with magnitude processing, and that these domain general processes also allow coding of serial order information in STM tasks. In sum, these different studies indicate that the horizontal segment of the IPS may support domain general ordinal processing shared with magnitude processing, and that these domain general processes also allow coding of serial order information in STM tasks.

The aim of the present study is to provide direct evidence for the existence of shared coding processes in the IPS for STM, numerical and alphabetical processing domains, by comparing distance effects in a serial order STM recognition task, a numerical order comparison task and an alphabetical order comparison task in which the common processes are the comparison and judgment of ordinal information through the assessment of the commonality of neural substrates associated with distance effects across tasks differing at the level of design and nature of stimuli, providing a strong test of our hypothesis. Although previous studies and models [15] suggest the possibility of overlapping neural substrates for processing information in these three domains, it still remains to be shown that identical neural activation patterns are actually involved when processing information in numerical, alphabetical and STM domains. Critically, no direct comparison as so far has been conducted between ordinal processing in STM and other domains, and this at both neural and cognitive levels; subsequently we do not know whether ordinal processing in STM and other domains is supported by exactly the same processes and neural substrates, or whether processing of serial order information in STM is supported by additional and distinct neural substrates.

We explored commonalities and differences at neural level in the same participants when they achieved an order STM, alphabetical order judgment and numerical judgment tasks. With regards to the coding representation, using a parametric design, we determined to what extent the anterior part of the horizontal segment of the IPS supports distance effects when comparing two numbers, two letters or two STM positions. In order to rule out that overlap in parietal areas across the different conditions could simply be due to increased difficulty and attentional requirements when judging information from close distances, we also focused on a more posterior part of the IPS which is known to be associated with enhanced attentional processing during STM tasks [27], [28]. Furthermore we included a control condition involving the judgment of luminance, with visual stimuli differing strongly or minimally at the level of luminance, thus mimicking distance processing but without manipulating any ordinal dimension of processing. The luminance judgment condition [24] was chosen and designed to control for basic visual perception/encoding processes as well as for comparison and decision processes, but without implying ordinal processing and comparison processes which are the critical component of the STM and alphabetical judgment events.

## Methods

### Ethics Statement

All participants gave their written informed consent to take part in the study, in line with the Declaration of Helsinki, and the study was approved by the Ethics Committee of the Medical School of the University of Liège.

### Participants

Twenty-six right-handed native French-speaking young adults (15 women), with no diagnosed psychological or neurological disorders, were recruited from the university community. The study was approved by the Ethics Committee of the Faculty of Medicine of the University of Liège, and was performed in accordance with the ethical standards described in the Declaration of Helsinki (1964). All participants gave their written informed consent prior to their inclusion in the study. Age ranged from 18 to 28 years, with a mean of 20.9 years. Minimal number of years of education was 14.

### Material

**Figure 1:**
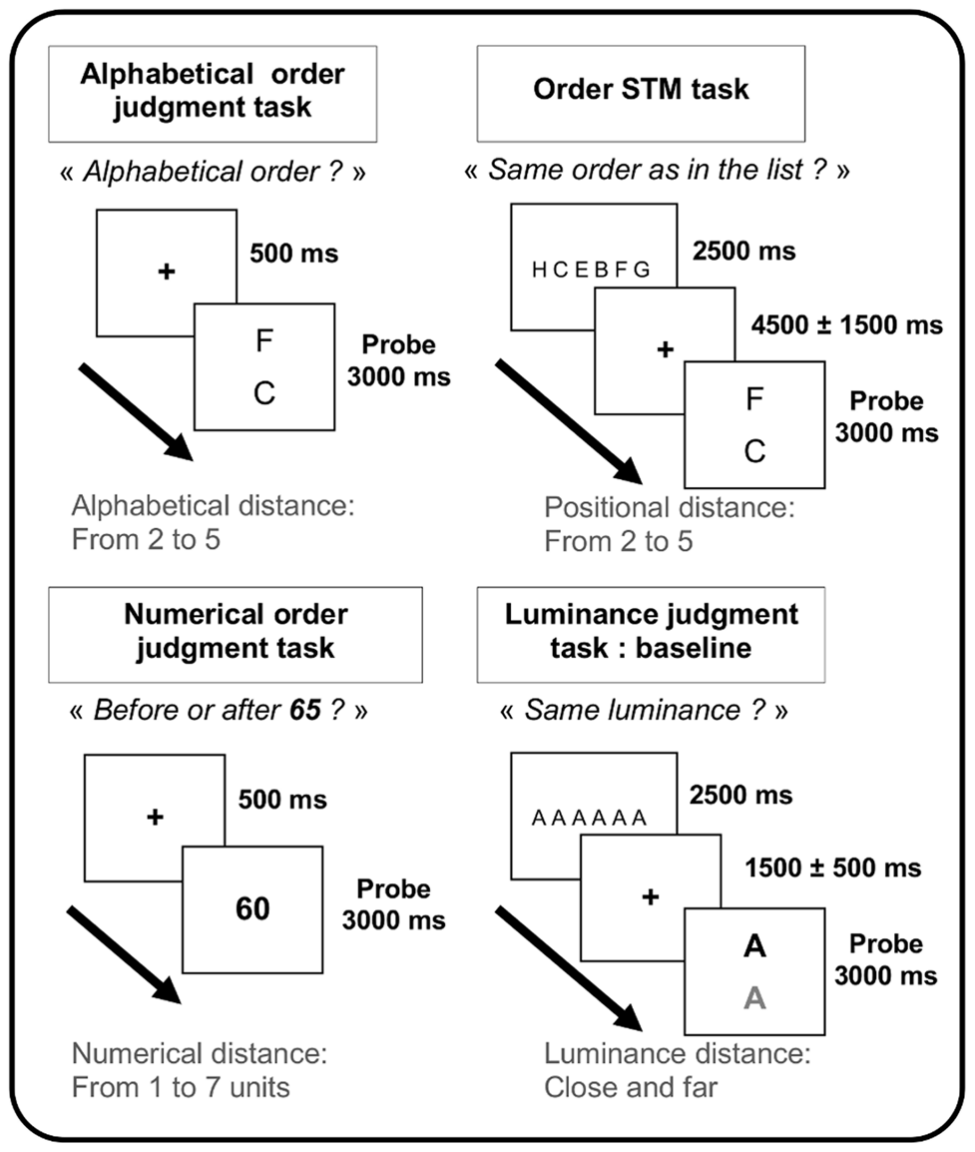
Experimental design and timing of the four tasks. For each condition, a negative probe trial is illustrated.

In a first fMRI session, the order STM and alphabetical order judgment conditions were administered, including also the luminance judgment control condition. For each STM trials, the encoding phase consisted of the presentation of a list of six letters (e.g., ‘D, C, I, F, J, A’) ordered horizontally (fixed duration: 2500 msec) (see also Figure 1). For the maintenance phase, a fixation cross was displayed for a variable duration (random Gaussian distribution centered on a mean duration of 4500±1500 msec). Finally, the retrieval phase consisted of an array of two probe stimuli ordered vertically, in order to eliminate the possibility that the task could be completed by mere visuo-spatial matching between the target and probe stimuli. Participants indicated within 3000 msec if order information for the two probe stimuli matched information in the memory list (by pressing the button under the middle finger for ‘yes’ and by pressing the button under the index for ‘no’). More specifically, participants judged whether the probe letter presented on the top of the screen had occurred in a more leftward position in the memory list than the probe letter presented on the bottom of the screen. The positional distance varied from 2 to 5, while keeping alphabetical distance constant (distance of 3). We used letters from A to I which were also the same letters used in the alphabetical order judgment task. In the alphabetical order judgment task, the participants saw two letters displayed vertically on the screen and they had to decide within 3000 msec whether they were displayed in correct alphabetical order. The participants responded by pressing the button under their index finger for ‘no’ responses and the button under their middle finger for ‘yes’ responses. The distances varied from 2 to 5 alphabetical positions. In order to favor automatic access to ordinal information, only the first nine letters of the alphabet were used. The number of distances was mainly determined by the STM task where we used a STM load of 6 items, which is known to challenge STM capacities without leading to floor effects [8], [27]. We did not assess distances of 1 position since several studies had observed a reverse distance effect for alphabetical order judgment [29], [30] as well as for order STM judgments especially for consecutive pairs in ascending order [9]. The luminance judgment control condition [24] consisted of the presentation of two identical letters (‘A’) displayed in white font on a black screen at identical or different luminance levels. The participants had to decide within 3000 msec whether luminance levels were the same or not by pressing the button under their index finger for ‘no’ and the button under their middle finger for ‘yes’. We manipulated two different luminance levels (close versus further apart) in the luminance baseline condition in order to control for general executive processes associated with comparison judgments for highly similar versus dissimilar stimuli in the tasks-of-interest, and hence to isolate neural substrates associated with ordinal judgments and comparison processes as specifically as possible. The photometric luminance difference between the two letters was either small (close distance = difference of 80 cd/m^2^ hue–saturation– brightness) or large (far distance = difference of 160 cd/m^2^ hue-saturation-brightness). In order to control for processes associated with basic letter processing in the STM task, the luminance judgment trials were furthermore preceded by the presentation of six identical letters (‘A’) organized horizontally and which the participants viewed passively (see Figure 1).

Finally, the neural substrates associated with ordinal distance effects for numerical information were assessed in a second session, using a numerical order judgment task based on the seminal paradigm developed by Pinel et al. [21]. After a fixation cross (250 msec), participants were presented a number (e.g., ‘45’; duration 1000 msec) and had to judge whether the number comes before or after the numerical standard ‘65’; the same standard was used for all trials [21]. The participants pressed on the button under the index finger for before-standard responses, and on the button under the middle finger for after-standard responses. The distances between the probe and the standard ranged from 1–7 units [distances 1 (60–64 and 66–70), 2 (55–59 and 71–75), 3 (50–54 and 76–80), 4 (45–49 and 81–85), 5 (40–44 and 86–90), 6 (35–39 and 91–95), and 7 (30–34 and 96–99)]. Given the relative long duration of the tasks administered in the first session, they were always presented first, and the numerical task was always presented in the second session, in order to diminish fatigue effects and to increase task compliance.

For the order STM and alphabetical judgment conditions there were 24 trials per ordinal distances. For the luminance judgment control condition, there were 20 trials by distance. For the numerical order judgment task there were 20 trials for distances 1 to 6 and 18 trials for the distance 7. For each condition and distance, there was an equal number of trials requiring a ‘yes’ or ‘no’ response. For each session and condition, the different trials were presented in pseudorandom order, with the restriction that 2 successive trials of the same distance and condition could not be separated by more than 5 trials of a different condition (i.e., by more than 65 s on average) in order to keep blood oxygen level dependent (BOLD) signals for same condition epochs away from the lowest frequencies in the time series. Before the start of a new trial, a cue informing about task condition appeared on the top of the screen during 1000 msec. The duration of the intertrial interval was variable (random Gaussian distribution centered on a mean duration of 2000±500 msec) and further varied as a function of the participants’ response times: the probe array disappeared immediately after a response was recorded. If the participant did not respond within 3000 msec, “no response” was recorded and the next trial began. Both response accuracy and response times were collected. Finally, a practice session outside the magnetic resonance environment, prior to the start of the experiment, familiarized the participants with the specific task requirements and included the administration of 10 practice trials.

### MRI Acquisition

Data were acquired on a 3-Tesla scanner (Siemens, Allegra, Erlangen, Germany) using a T2*-sensitive gradient-echo EPI sequence (TR = 2040 msec, TE = 30 msec, field of view (FOV) = 192×192 mm^2^, 64×64 matrix, 3 mm in-plane resolution, 34 axial slices with 3 mm thickness, and 25% interslice gap to cover most of the brain. The 3 initial volumes were discarded to avoid T1 saturation effects. Field maps were generated from a double-echo gradient recalled sequence (TR = 517 msec, TE = 4.92 and 7.38 msec, FOV = 230×230 mm^2^, 64×64 matrix, 34 transverse slices with 3 mm thickness and 25% gap, flip angle = 90°, bandwidth = 260 Hz/pixel) and used to correct echo-planar images for geometric distortion due to field inhomogeneities. A high-resolution T1-weighted magnetization-prepared rapid gradient echo image was acquired for anatomical reference (TR = 1960 msec, TE = 4.4 msec, time to inversion = 1100 msec, FOV = 230×173 mm^2^, matrix size 256×192×176, voxel size 0.9×0.9×0.9 mm^3^). For the first session (STM, alphabetical and luminance judgment), between 1134 and 1272 functional volumes were obtained. For the second session (numerical order judgment), between 346 and 392 functional volumes were obtained. Head movement was minimized by restraining the subject's head using a vacuum cushion. Stimuli were displayed on a screen positioned at the rear of the scanner, which the subject could comfortably see through a mirror mounted on the standard head coil.

### fMRI Analyses

Data were preprocessed and analyzed using SPM8 software (Wellcome Department of Imaging Neuroscience, www.fil.ion.ucl.ac.uk/spm) implemented in MATLAB (Mathworks, Natick, MA). EPI time series were corrected for motion and distortion using “Realign and Unwarp” [31] using the generated field map together with the Fieldmap toolbox [32] provided in SPM8. A mean realigned functional image was then calculated by averaging all the realigned and unwarped functional scans, and the structural T1 image was coregistered to this mean functional image (rigid body transformation optimized to maximize the normalized mutual information between the 2 images). The mapping from subject to Montreal Neurological Institute space was estimated from the structural image with the “unified segmentation” approach [33]. The warping parameters were then separately applied to the functional and structural images to produce normalized images of resolution 2×2×2 mm^3^ and 1×1×1 mm^3^, respectively. The scans were screened for motion artifacts and time series with movements exceeding 3 mm (translation) or 3° (rotation) were discarded; this resulted in the removal of the data of 2 participants not presented here. Finally, the warped functional images were spatially smoothed with a Gaussian kernel of 8 mm full-width at half maximum (FWHM).

For each subject brain responses were estimated at each voxel, using a general linear model with epoch regressors and event-related regressors. For the order STM and luminance judgment conditions, regressors were defined to cover encoding, maintenance and retrieval phases. Encoding and maintenance phases were modeled via a single regressor due to the short duration of the encoding phase leading to high autocorrelation between these two phases. The encoding-maintenance regressor ranged from the onset of each trial until the onset of the probe display. On this basis, we obtained two linear contrasts corresponding to the encoding-maintenance phase of order STM and luminance judgment conditions. For the order STM retrieval stage, as well as the alphabetical ordinal judgment, the luminance judgment retrieval stage and the numerical order judgment conditions, the regressor ranged from the onset of the probe display to the participant’s response. In order to ensure minimal autocorrelation between the two phase-specific regressors, the encoding/maintenance regressors for luminance and order STM was further orthogonalized relative to the other two retrieval regressors [4], [5], [10], [27], [34]: Shared variance between retrieval and late encoding/maintenance phases was attributed to the retrieval regressor. On this basis, for each condition, one linear contrast was performed, one for the encoding/maintenance phase of order STM, one for the retrieval phase of order STM and one for the alphabetical order judgment; for each of these contrasts, the corresponding luminance baseline events were subtracted.

After that, for each subject and each condition, a parametric design was defined in order to highlight voxels sensitive to ordinal distance effects. For the order STM retrieval phase, the ordinal letter judgment, the luminance judgment and the numerical order judgment tasks, the regressor ranged from the onset of the probe display to the participant’s response with a parametric modulation for each distance. For each parametric design, the model included a regressor looking at activation whose intensity was modulated linearly by numerical/positional/alphabetical/luminance distance, plus their time derivatives. This model was applied for each task condition resulting in one target contrast for each condition. These contrasts were then entered in second-level analyses, corresponding to random effects models. One-sample t-tests for each phase of the STM, as well as for the alphabetical order judgment and the numerical order judgment were used to identify cerebral correlates of each condition for the task-related and distance effects. Null conjunction analyses assessed the commonality of activation profiles associated with the parametric distance effects across the different tasks [35] by exclusively masking for activation due to the luminance distance effect condition but also the brain activation differences between the each condition.

For each model, the design matrix also included the realignment parameters to account for any residual movement-related effect. A high-pass filter was implemented using a cutoff period of 128 sec in order to remove the low-frequency drifts from the time series. Serial autocorrelations were estimated with a restricted maximum likelihood algorithm with an autoregressive model of order 1 (+ white noise). The resulting set of voxel values constituted a map of t statistics [SPM(t)]. All contrast images were then smoothed again (6-mm FWHM Gaussian kernel) in order to reduce remaining noise due to intersubject differences in anatomical variability in the individual contrast images. Statistical inferences were performed at the voxel level at *p*<.05, with FWE- corrections for multiple comparisons across the entire brain volume, as well as using small volume corrections for a priori locations of interest [36].

### A Priori Locations of Interest

Regions of interest concerned the anterior and posterior bilateral IPS, based on aforementioned studies associating the anterior part of the horizontal of the IPS to processing of ordinal information in STM and numerical processing tasks, and associating the posterior part of the IPS to attentional control processes. The small volume correction was computed on a 10 mm radius sphere around the averaged coordinates published for the corresponding location of interest namely bilateral anterior IPS (−42, −42, 40 and 44, −40, 44) [4], [5], [10], [24], [37] and bilateral posterior IPS (−26, −62, 46; 28, −58, 40) [27], [28].

## Results

### Behavioral Data

**Figure 2:**
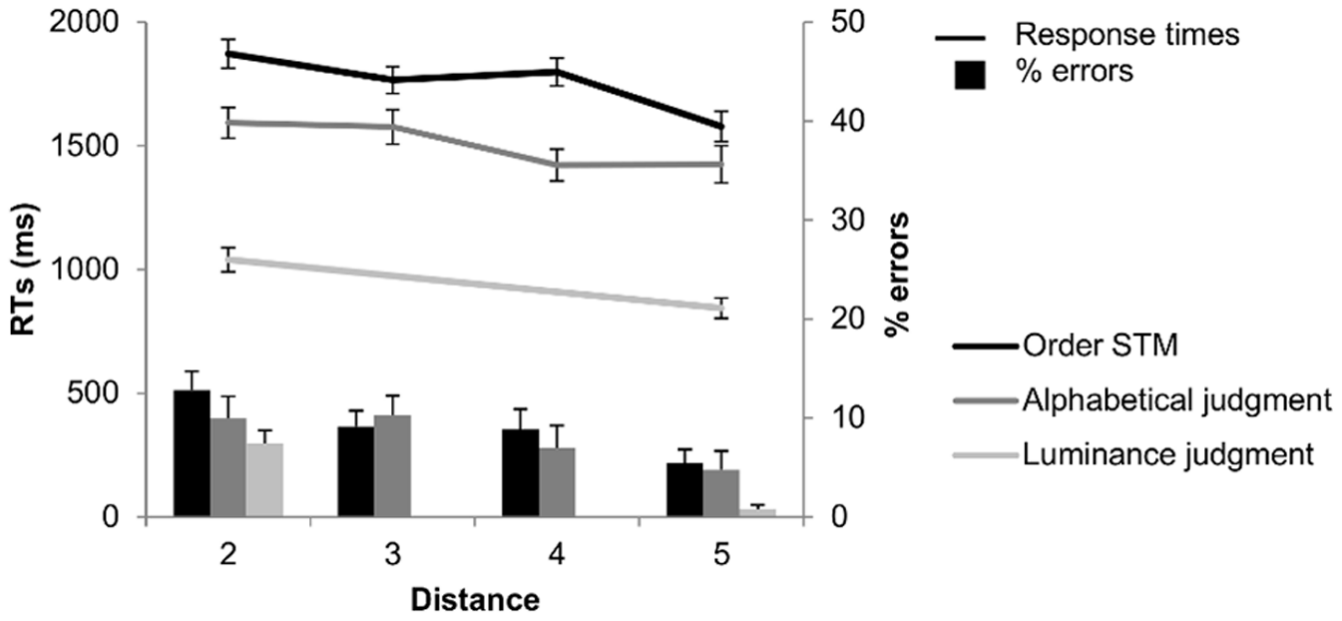
Response times and errors percentage for the order judgment tasks. Behavioral performances in order STM, alphabetical judgment, and luminance judgment tasks, as a function of positional, alphabetical and luminance distance, respectively.

**Figure 3:**
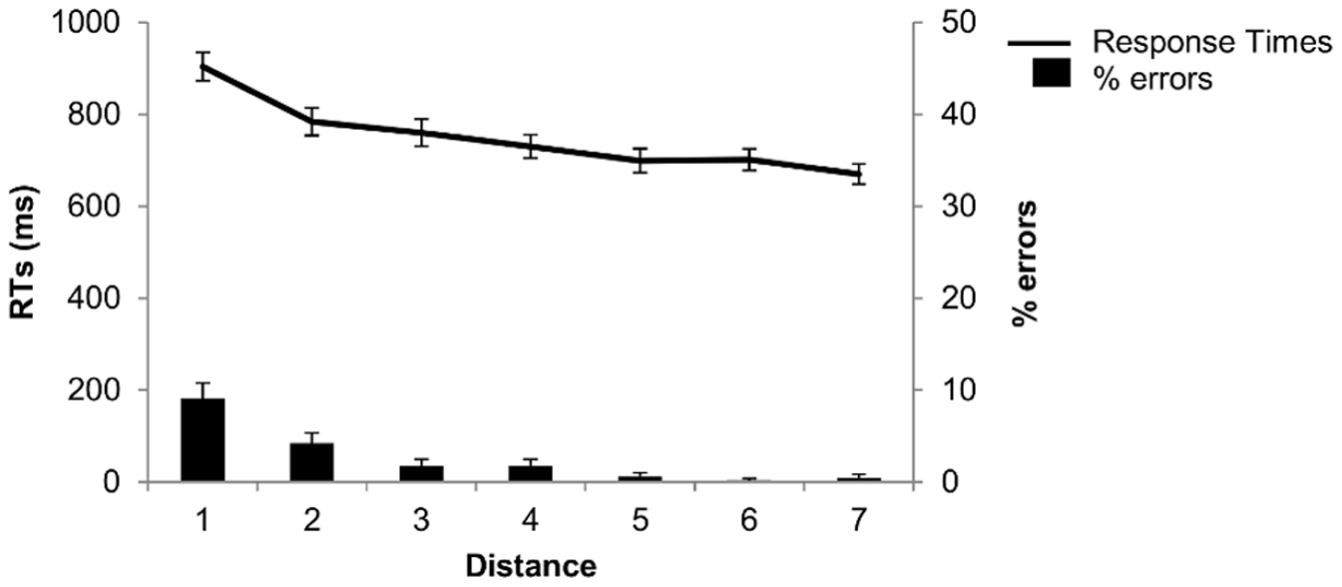
Response times and errors percentage for the numerical comparison task. Behavioral performances in number comparison task, as a function of numerical distance.

General behavioral results are shown in Figure 2 and 3. Although tasks were closely matched for task difficulty as indicated by the overall high performance levels across tasks, there was nevertheless an advantage for the luminance and numerical order judgment tasks (STM: error rate = 9%; alphabetical order judgment: error rate = 8%; luminance: error rate = 4%; numerical order judgment: error rate = 3%). A one-way ANOVA with task as repeated measures showed a main effect of task (*F*(3,75) = 8.69; η^2^ = 0.26; *p<*0.001). Planned comparisons showed significant differences only between the two first conditions (STM and alphabetical order judgment) and the two other conditions (luminance and numerical order judgment) (all Ps <.05). Response times (RTs) followed the same pattern with mean RTs larger in STM (1723 msec) and in alphabetical order judgment conditions (1504 msec) than in the luminance condition (968 msec) and the numerical order judgment task (749 msec). A one-way ANOVA on RTs with task as repeated measures showed also a main effect of task (*F*(3,75) = 231.48; η^2^ = 0.90; *p<*0.001). Planned comparisons showed significant differences between all tasks (all Ps <.001). In sum, order STM and alphabetical judgment conditions were slightly more difficult to perform than the number comparison task and the luminance judgment condition. These results are not surprising since alphabetical sequence knowledge is likely to be less automatic than numerical sequence knowledge [38], and for the order STM condition, order probe recognition requires retrieval of the memoranda of the memory list.

The crucial measure for this study concerned the distance effects. Accuracy and reaction times (RTs) were analyzed as a function of distance using one-way ANOVAs with distance as repeated measures. For the order STM condition, we observed a main effect of distance for accuracy (*F*(3,75) = 4.09; η^2^ = 0.14; *p<*0.001) and RTs (*F*(3,75) = 31.83; η^2^ = 0.56; *p<*0.001). Planned comparisons showed a significant difference between distance 2 and distance 5 for accuracy and between all distances (Ps <.05) except distances 2 vs. 4 and distances 3 vs. 4 for RTs. For the alphabetical order judgment condition, we also observed a main effect of distance for accuracy (*F*(3,75) = 3.65; η^2^ = 0.13; *p<*0.05) and RTs (*F*(3,75) = 19.36; η^2^ = 0.44; *p<*0.001). Planned comparisons showed significant differences for distances 2 vs. 5 and distances 3 vs. 5 (Ps <.05) for accuracy, and between all distances for RTs (all Ps <.001) except distances 2 vs. 3 and distances 4 vs. 5 for RTs. For the luminance judgment condition, we also observed a significant distance effect for accuracy (*F*(1,25) = 19.07; η^2^ = 0.43; *p<*0.001) as well as RTs (*F*(1,25) = 73.36; η^2^ = 0.75; *p<*0.001). Finally, the same was true for the numerical order judgment task, with a significant distance effect for accuracy (*F*(6,150) = 16.95; η^2^ = 0.40; *p<*0.001) and RTs (*F*(6,150) = 56.52; η^2^ = 0.69; *p<*0.001). For accuracy, planned comparisons showed significant differences between all distances except for the most contiguous ones (3 vs. 4, 4 vs. 5, 5 vs. 6 and 6 vs. 7) (all Ps <.05). For RTs, the same was true with significant differences between all distances except for the most contiguous ones (2 vs. 3, 4 vs. 5, 5 vs. 6 and 6 vs. 7) (all Ps <.05). Overall, we observed the expected distance effects for all tasks, with the strongest effects for RT’s.

We also determined the intercorrelations between the different behavioral distance effects. We computed for each participant the size of the behavioral distance effect by subtracting the RTs for the shortest distance from those of the longest distance, and by dividing this result by the sum of the two RTs [39], [40]. The size of the distance effects correlated significantly between all task conditions (order STM and alphabetical order judgment: r = .57; p<.01; order STM and numerical ordinal judgment: r = .51; p<.01; alphabetical and numerical order judgment: r = .46; p<.05). However, when controlling via a partial correlation for the luminance judgment distance effect, only the correlation between distance effects for the order STM and alphabetical order judgment remained significant (r = .55; p<.01); the correlation with numerical order judgment became non-significant (order STM and numerical ordinal judgment: r = .03; p>.05; alphabetical and numerical order judgment: r = .11; p>.05). These data suggest that distance effects arise from identical processes in the STM and alphabetical conditions, presumably related to ordinal processing. For the numerical condition, distance effects may arise from multiple levels, including ordinal as well as magnitude processing levels.

### Imaging Data

#### Task-related effects

We computed one-sample t-tests to determine the overall activation patterns for the retrieval phase during the order STM condition, for alphabetical order judgment and numerical order judgment conditions. First, for the retrieval phase during order STM, we observed activations in the left precentral gyrus, the left superior and inferior frontal gyrus, the left superior parietal gyrus, the left anterior IPS, the right calcarine sulcus, the left lingual gyrus and the right cerebellum. Retrieval of information in the order STM was thus associated with enhanced activation in dorso- and ventro-lateral prefrontal regions, in line with previous studies involving maintenance and retrieval of information in STM [4], [9], [27]. For the alphabetical order judgment, we observed activations in the right calcarine sulcus, the left lingual and the right caudate nucleus; these activations are also in line with previous studies that used the same type of alphabetical comparison task [25]. Overall, these two tasks activated the expected networks relative to the previous studies using the same type of tasks; importantly, these activations are associated specifically with these tasks and do not reflect general processes involved in stimulus comparison and response decision processes since these were controlled via the luminance condition. Finally, for the numerical order judgment task, we observed activations in the left postcentral gyrus, the bilateral anterior IPS, the left insula and the left putamen again in line with previous literature (see Table 1).

**Table 1:** Maxima within regions showing BOLD signal changes in the retrieval phases of order STM, the alphabetical order judgment and the numerical order judgment task-related effects and common sustained activation peaks (null conjunction) between task-related effects.

		*Order STM retrieval*						*Alphabetical order judgment*							*Numerical order judgment*						
Anatomical region	No.voxels		x	y	z	SPM (Z)-value	BAarea	No.voxels		x	y	z	SPM (Z)-value	BAarea	No.voxels		x	y	z	SPM (Z)-value	BAarea
Precentral	56	L	−50	0	46	5.09	6														
Postcentral															28	L	−52	−20	16	4.95	48
Superior frontal gyrus	35	L	−28	6	70	5.15	6														
Inferior frontal gyrus	545	L	−36	18	26	5.38	48														
Superior parietal gyrus	969	L	−24	−70	42	5.92	7														
Anterior IPS	60	L	−34	−46	38	3.95*	40								205	L	−50	−34	52	5.17	40
															1112	R	36	−56	−36	5.56	40
Insula															38	L	−40	−6	10	4.93	48
Calcarine sulcus	168	R	14	−88	2	5.76	17	426	R	14	−88	2	6.10	17							
Lingual gyrus	324	L	−14	−92	−10	5.50	18	768	L	−14	−84	−22	6.37	18							
Caudate								42	R	16	0	26	4.91								
Putamen															122	L	−22	−4	10	5.30	
Cerebellum	406	R	30	−68	−28	5.72															
***Alphabetical order judgment ∩ Order STM ∩ Numerical order judgment***																					
No suprathreshold voxels																					
***Alphabetical order judgment ∩ Order STM***																					
Calcarine sulcus	153	R	14	−88	2	5.76	17														
Lingual gyrus	165	R	12	−80	−18	5.53	18														
	320	L	−14	−92	−10	6.49	18														
***Order STM ∩ Numerical order judgment***																					
Cerebellum	129	R	32	−68	−30	5.58															
***Alphabetical order judgment ∩ Numerical order judgment***																					
No suprathreshold voxels																					

Note: If not otherwise stated, all regions are significant at p<.05, corrected for whole brain volume.

*p<.05, small volume corrections.

Next, we determined the commonality of activations associated with the different task-related effects. First, we conducted a null conjunction analysis over the task-related effects for the order STM, alphabetical and numerical processing tasks. This analysis did not show common neural network across tasks (see Table 1). In order to more fully understand this result, we conducted further pair-wise null conjunctions. A null conjunction analysis over the distance effect in the order STM and alphabetical processing tasks showed a common involvement of the right calcarine sulcus and the bilateral lingual sulcus (see Table 1). For the order STM and the numerical order judgment tasks, conjunction analysis revealed common involvement of the right cerebellum; the null conjunction between the alphabetical and numerical order judgment tasks did not show common overlapping activations (see Table 1). In sum, although the different tasks yielded the expected activations patterns, overall task-related activations differed between the three tasks.

### Parametric Analyses

**Figure 4:**
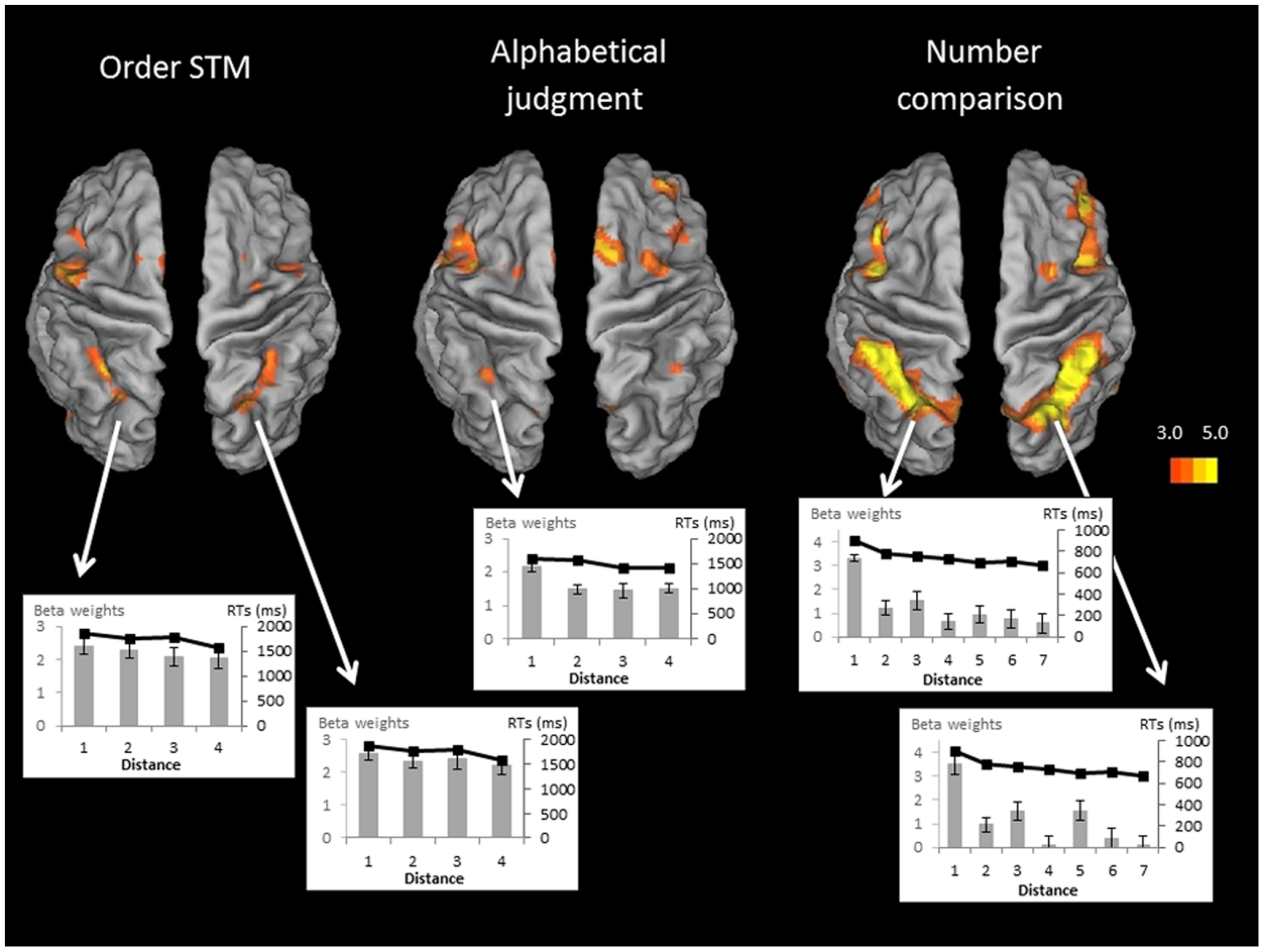
Brain to the distance effect in order STM, alphabetical judgment and number comparison conditions. Regions are shown with a display threshold of 3≤ Z ><5. The results are mapped onto an inflated brain template using Caret 5.64 with the PALS-B12 atlas [63], [64]. Brain areas presenting a strictly monotonic decrease of percentage activation (grey columns) with positional/alphabetical distance (p<.001, uncorrected) similar to the pattern of reaction times (black curve). Data are averaged across conditions.

First, we examined neural correlates associated with distance effects for each task condition. When judging the order of two items held in STM, the participants showed significant modulation of brain activity as a function of positional distance in the bilateral IPS and this in both anterior and posterior parts of the bilateral IPS (see Table 2 and Figure 4). At a lower statistical threshold (*p* = .001, uncorrected), we also found activation in the left precentral gyrus, left thalamus and middle cingulum areas (respectively, MNI coordinates: −54, 4, 38, *Z* = 4.09; −8, −8, 4, *Z* = 4.02; 6, 20, 46, *Z* = 3.91) in line with previous studies [4], [9]. An analysis of mean beta values in the bilateral anterior IPS showed that activation decreases quasi-monotonically with increasing positional distance, paralleling the linear decrease of response times (see Figure 4). When considering the distance effect in the alphabetical order judgment task, very similar results were observed with activation in the bilateral IPS but only in the anterior part of the IPS, (see Figure 4). Mean beta values decreased quasi-monotonically with increasing alphabetical distance, paralleling again response times (see Figure 4). In addition, the distance effect was also associated here with activation in the supplementary motor area as in previous studies which have been associated with control and decision processes [24], [41], [42] (see Table 2). For the numerical order judgment task, results were again very similar, with bilateral anterior but not posterior IPS involvement as a function of numerical distance, characterized by decreasing activation with increasing distance (see Figure 4). Additional distance-sensitive activation was observed in the right inferior frontal gyrus (see Table 2). Again, mean beta values decreased quasi-monotonically with increasing numerical distance, paralleling response times (see Figure 4). Finally, the distance effect of luminance judgment control condition, although significant at the behavioral level, did not elicit significant brain modulation in target areas. However, at a less conservative threshold (p = 0.001, uncorrected), stronger activation in a fronto-occipital network was observed for closer luminous intensities (respectively, coordinates: 32, 32, 14 mm; *Z* = 3.41; −30, −84, −6 mm; *Z* = 3.26) reflecting stronger demands on visual processing and visual control [43].

**Table 2:** Functional activation peaks for the distance effects of order STM, alphabetical judgment, luminance judgment and numerical judgment tasks.

Anatomical region	No. voxels	Left/right	x	y	z	SPM (Z)-value	BA area
***Order STM***							
IPS anterior	25	L	−34	−48	40	3.50*	40
	105	R	44	−34	38	3.80*	40
			38	−40	42	3.50*	40
			40	−48	48	3.26*	40
IPS posterior	140	L	−28	−54	42	4.81*	7
			−26	−60	42	4.63*	7
	98	R	24	−62	44	3.70*	40
			34	−52	44	3.13*	40
***Alphabetical order judgment***							
SMA	25	L	−2	14	44	4.73	32/24
		R	4	18	52	4.76	8/6
IPS anterior	29	L	−38	−50	36	3.89*	40
	45	R	46	−40	36	3.55*	40
***Luminance judgment***							
	No suprathreshold voxels						
***Numerical order judgment***							
Inferior frontal gyrus (Orb)	22	R	30	22	−6	4.88	47/11
IPS anterior	82	L	−34	−52	40	5.19	40
	43	R	40	−48	42	4.98	40
IPS posterior	444	L	−34	−58	44	5.47*	7
			−30	−66	54	4.54*	7
	386	R	22	−66	44	4.67*	7
			34	−62	46	4.58*	7

Note: If not otherwise stated, all regions are significant at p<.05, corrected for whole brain volume.

*p<.05, small volume corrections.

Next, we determined the commonality of activations associated with distance effects across the different tasks and conditions. For all conjunction analyses, we checked that the overlap of neural activation was not driven by mere differences in task difficulty of the distances to be judged in controlling for any neural activity related to non-ordinal distance judgment by exclusively masking for activation due to distance effects in the luminance condition.

**Figure 5:**
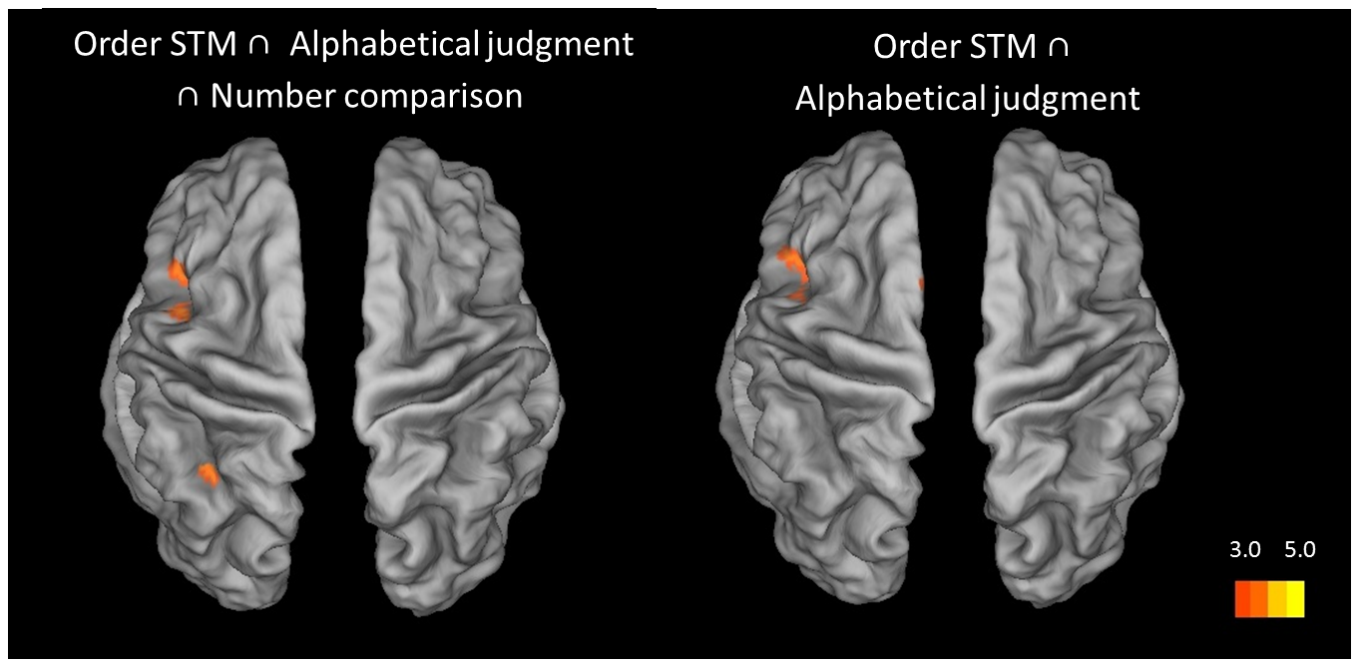
Brain to the conjunction between the distance effects of all conditions. Regions are shown with a display threshold of 3≤ Z ><5. The results are mapped onto an inflated brain template using Caret 5.64 with the PALS-B12 atlas [63], [64].

**Figure 6:**
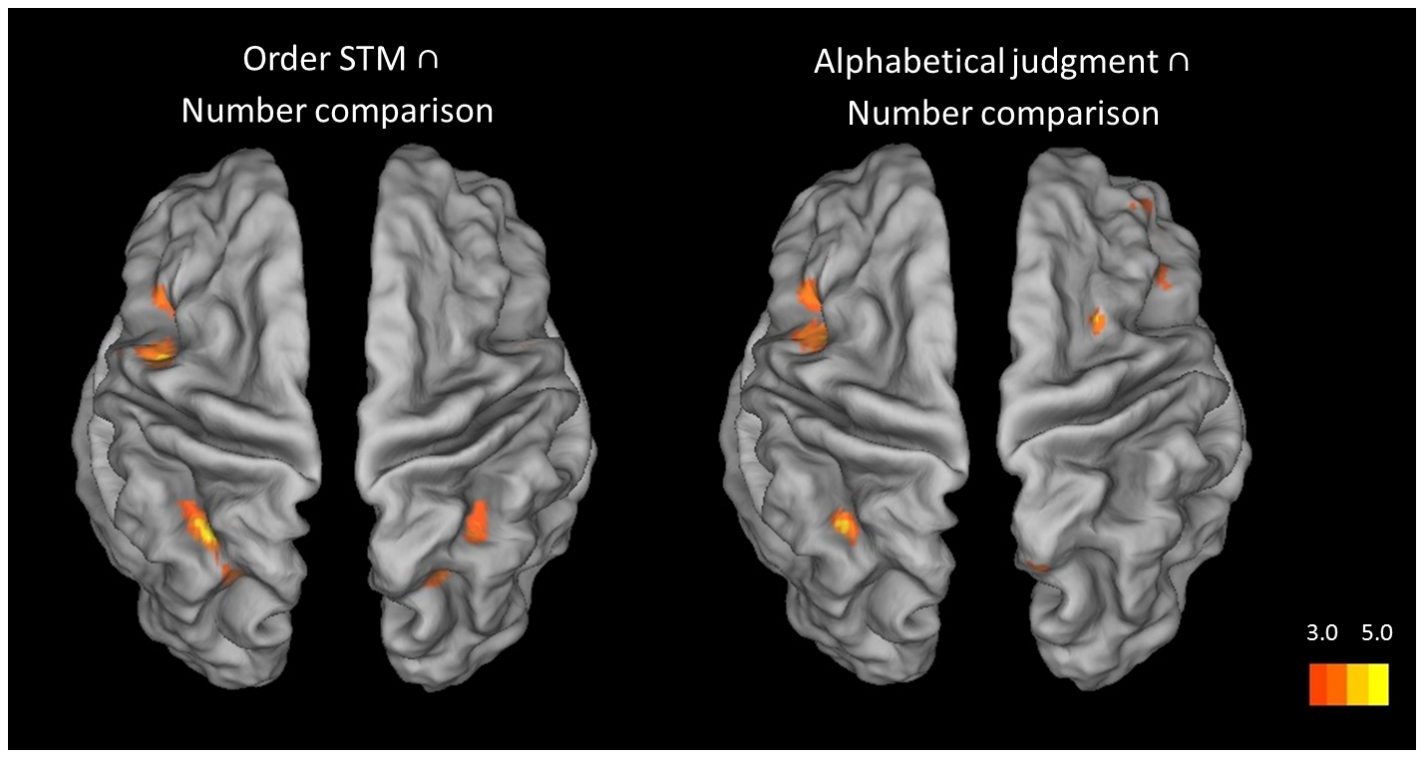
Brain to the conjunction between the distance effect of ordinal conditions and number comparison. Regions are shown with a display threshold of 3≤ Z ><5. The results are mapped onto an inflated brain template using Caret 5.64 with the PALS-B12 atlas [63], [64].

First, we conducted a null conjunction analysis over the distance effects for the order STM, alphabetical and numerical processing tasks. This analysis showed a common involvement of the left anterior IPS only (see Table 3 and Figure 5). In order to more fully understand this result, we conducted further pair-wise null conjunction. A null conjunction analysis over the distance effect in the order STM and alphabetical processing tasks showed again common activation restricted to the left anterior IPS (see Table 3 and Figure 5). For the order STM and the numerical order judgment tasks, however, conjunction analysis revealed additional overlapping involvement of the bilateral anterior and posterior IPS regions. Finally, for the null conjunction over the distance effect in the alphabetical and numerical order judgment tasks, overlapping activation was observed in the left anterior IPS region, and to lesser extent in the right anterior IPS region (see Table 3 and Figure 6). In sum, the distance effects for the order STM and alphabetical order judgment conditions recruited the same left anterior IPS regions as the distance effect in the numerical judgment task. Although the right anterior IPS showed less consistent commonality over the three conditions, differential effect analyses by contrasting the three distance effects on a pair-wise basis, revealed no significant differences in the right IPS, nor in posterior IPS target regions, between the three conditions. In the single effect analysis, the right aIPS was also sensitive to distance in the alphabetical order judgment task, but the peak of activation was slightly more lateral and lower on the z-axis than in the two other two conditions, explaining the less consistent results for this region in the null conjunction analyses which are very conservative at the statistical level.

**Table 3:** Common sustained activation peaks (null conjunction) for the positional distance effects of order STM, alphabetical order judgment and numerical order judgment tasks with control of luminance judgment.

Anatomical region	No. voxels	Left/right	x	y	z	SPM (Z)-value	BA area
***Alphabetical order judgment ∩ Order STM ∩ Numerical order judgment***							
IPS anterior	19	L	−36	−50	40	3.77*	40
***Alphabetical order judgment ∩ Order STM***							
IPS anterior	3	L	−36	−50	40	3.34*	40
***Order STM ∩ Numerical order judgment***							
IPS anterior	81	L	−34	−48	40	3.93*	40
	102	R	44	−36	38	3.90*	40
			38	−48	44	3.31*	40
IPS posterior	145	L	−30	−54	42	4.22*	7
	135	R	24	−62	42	4.07*	7
			34	−52	42	3.56*	7
***Alphabetical order judgment ∩ Numerical order judgment***							
IPS anterior	40	L	−38	−50	38	3.98*	40
	19	R	46	−40	36	3.38*	40

Note: If not otherwise stated, all regions are significant at p<.05, corrected for whole brain volume.

*p<.05, small volume corrections.

### Phase-specific STM Activations

The final analysis was designed to check for the involvement of distance-sensitive IPS regions during the encoding and maintenance stages of the STM task in order to show that distance-sensitive IPS involvement in the STM tasks does not only originate from comparison processes during retrieval but also supports encoding and maintenance of serial order information in STM. We computed one sample *t* tests to determine overall activation patterns during encoding and maintenance (see Table 4). During the encoding/maintenance phase, activation of a fronto-parieto-temporo-cerebellar network was observed to be activated, including bilaterally, the posterior IPS. In the left hemisphere, the supplementary motor area, the postcentral gyrus, the superior frontal gyrus, the anterior IPS, the middle temporal gyrus, the inferior occipital gyrus and the hippocampus and, in the right hemisphere, the middle frontal gyrus, the lingual gyrus and the cerebellum were activated (see Table 4). We examined whether the neural substrates supporting the distance effects during retrieval were also involved during the encoding-maintenance STM phase by using the activations of the distance effect conjunction analyses as an inclusive mask. When using the neural substrates associated with the three distance effects (conjunction analyses) as an inclusive mask, we observed overlap of activation in the left precentral gyrus, the left inferior frontal gyrus and the left anterior IPS. Finally, overlap of activation was observed in the left precentral gyrus, the left inferior frontal gyrus, the left anterior IPS and the bilateral posterior IPS when using an inclusive mask for neural substrates associated with distance effects in the STM and numerical domains only (see Table 4). These results suggest that the left anterior IPS area which is sensitive to ordinal distance information during retrieval is also supporting encoding and maintenance of order information.

**Table 4:** Functional activation peaks for the encoding-maintenance phase of order STM tasks.

Anatomical region	No. voxels	Left/right	x	y	z	SPM (Z)-value	BA area
***Order STM : encoding-maintenance phase***							
SMA	125	L	−2	−2	74	5.65	6
Postcentral gyrus	2084	L	−56	−2	20	5.95	43/48
Superior frontal gyrus	1214	L	−4	−48	−22	5.81	21
Middle frontal gyrus	134	R	42	36	30	5.44	46
IPS anterior	255	L	−36	−42	32	4.32*	40
IPS posterior	597	L	−24	−70	44	5.82	7
	283	R	32	−70	50	5.42	7
Middle temporal gyrus	166	L	−66	−28	2	5.81	21
Inferior occipital gyrus	67	L	−50	−64	−14	5.78	37
Hippocampus	1545	L	−24	−18	−12	6.75	
Lingual gyrus	58	R	24	−94	−10	5.56	
Cerebellum	1797	R	28	−70	−30	6.36	
***Encoding-maintenance phase with inclusive mask over neural substrates associated with the three distance effects***							
Precentral gyrus	91	L	−42	8	26	5.25	48
Inferior frontal gyrus	55	L	−44	20	24	5.04	48
IPS anterior	19	L	−36	−46	34	4.78	40
***Encoding-maintenance phase with inclusive mask over neural substrates associated with distance effects in STM and numerical conditions***							
Precentral gyrus	108	L	−42	8	26	5.25	48
Inferior frontal gyrus	51	L	−44	20	24	5.04	48
IPS anterior	62	L	−36	−44	34	4.14*	40
IPS posterior	119	L	−24	−68	44	5.76	7
	19	R	22	−66	48	4.99	7

Note: If not otherwise stated, all regions are significant at p<.05, corrected for whole brain volume.

*p<.05, small volume corrections.

## Discussion

The present study tested the hypothesis that anterior IPS involvement during STM tasks is related to domain-general ordinal coding processing, supporting serial order coding in STM, but also ordinal representation of numerical and alphabetical information. While several studies [9], [15], [16], [21], [24] suggest the possibility of common neural substrates involved in processing ordinal information across different domains, none has investigated these commonalities directly and within the same participants, and this particularly for the STM domain as opposed to numerical or alphabetical domains. We observed that the positional distance effect in an order STM probe recognition task, and the ordinal distance effects in alphabetical order judgment and numerical order comparison tasks showed common involvement of the anterior part of the horizontal segment of the left IPS. In addition, common distance-sensitive activation was also observed in the right anterior IPS for the order STM task and the numerical order judgment task. These commonalities were also confirmed by behavioral results showing an intercorrelation of the size of the behavioral distance effect between the three tasks.

The present study demonstrates that the parietal lobe, and more specifically the anterior part of the IPS, plays a critical role in order processing across different domains, such as STM, letter knowledge and numerical cognition. These are, to the best of our knowledge, the first empirical data to show directly that order processing in STM engages identical regions to those supporting order processing of alphabetic and numerical information. More precisely, the present data show that the neural substrate located in the anterior part of the IPS presents increasing activation for more fine-grained distance discriminations. It is important to note here that these distance-sensitive activations cannot be simply ascribed to a greater attentional involvement for difficult (close position) trials, since we controlled for this possibility via the luminance judgment condition, for which no aIPS activation was observed. Also, we did not observe common posterior IPS activation, which is known to be associated with enhanced attentional processing during STM tasks [27], [28], [44].

As mentioned in the Introduction, although there are many different models of serial order coding in STM, all rely on the basic implicit assumption that serial order coding requires some form of ordinal signal or activation gradient [7], [11]–[14], [45]. Some neural network model have linked order coding in STM in an explicit way to ordinal processing, by proposing that serial order information is coded using ordinal rank information shared with numerical cognition [15], [16]. These authors also pointed to the IPS as supporting the representation of these ordinal codes. Furthermore, neurons in the IPS also have been shown to respond selectively to the number of occurrences of a given event, with distinct neurons responding to the first, second, third, … event [46]. Altogether, these different findings suggest that ordinal coding is a basic property of serial order coding in STM and that this function is supported by the anterior part of the IPS.

Furthermore, the present study suggests that these ordinal coding processes are not only shared with number processing, but they also intervene for alphabetical order decisions. These results are in line with a number of studies showing similar distance effects for numerical and non-numerical judgment (numbers and letters: [24]; numbers and months: [19], [26]). Likewise, Fulbright et al. [25] observed activation of the IPS for tasks involving the ordering of numbers, size dimensions and letters. We should however note that Zorzi et al. [47] observed that within the bilateral horizontal segment of the IPS, multi-variate analysis techniques are able to discriminate between numerical and alphabetical order processing. In the same way, using an event-related potential paradigm, Szűcs and Csépe [48] revealed some similarities but also differences in the activation patterns for ordinal coding of numerical and non-numerical information. These results indicate that although the same intraparietal areas are recruited, ordinal information may nevertheless be associated with different neural dynamics in this region as a function of processing domain.

The different tasks used in this study were not perfectly matched in terms of distance, response or stimulus type. This was part of the rationale of the present study since it enabled us to provide a strong test of our hypothesis, by showing that distance effects across tasks and domains are supported by the same neural substrates, and that these commonalities are not just an artefact of very similar task designs for conditions-of-interest. Indeed, conjunction analyses for task-related effects revealed no common activations in fronto-parietal networks of interest here. Importantly, there were several differences between numerical judgment task and the order STM and alphabetical judgment tasks. The numerical judgment task was inspired by the original study reported by Pinel et al. [21] who were the first to highlight numerical distance effects in the bilateral IPS; in order to remain as close as possible to their task and findings as regards the numerical distance effect, we used their original task parameters which included 7 distances. However, due to capacity limitations for the STM task, we used STM lists of 6 items leading to a smaller number of distances that could be assessed during STM probe recognition trials; this was also true for the alphabetical condition, which was closely matched to the STM task. Furthermore, for the numerical task, a single number has to be judged relative to a single constant standard, while in the alphabetical comparison condition, the canonical order of two different, simultaneously presented letters has to be compared. Despite these differences, we observed the expected sensitivity of the IPS for distance effects across the three tasks, and this most particularly in the posterior IPS for the order STM and numerical tasks which maximally differed in terms of task design.

It is important to consider here the potential different roles of the left and the right aIPS in ordinal processing. In the present study, we observed that the left aIPS showed ordinal distance sensitivity in all three ordinal task conditions (STM, letters, numbers), while the right aIPS target area showed also distance sensitivity, but only consistently for the number and STM conditions. A number of studies have proposed that the left aIPS may exert a more abstract relational processing role, while the right aIPS appears to be more specifically associated with number processing, which is in line with the findings of the present study [23], [24], [49], [50]. For instance, processing of both numerical and non-numerical magnitude has been associated with left aIPS activation [51]–[55]. Some studies have also suggested that the left IPS supports more fine-tuned representations for symbolic and nonsymbolic quantities than the right [53], [56]–[58]. These data indicate that during order processing in STM tasks, two levels of ordinal representations may be involved, one more abstract shared with ordinal processing across a large number of domains, and one more directly related to number processing, using number rank information to code serial position in STM.

More generally, our data are in line with recent studies suggesting close connections between STM and numerical processing. Two types of conceptual frameworks currently co-exist. The one motivating the present study considers the existence of an ordinally organized representational system, at numerical and more abstract levels, whose representations are used to code serial order information in STM [14], [15]. A second framework however considers that ordinal representations do not exist per se, but are created temporarily in WM via spatial attention processes. van Dijck et al. [59] recently showed that retrieval of serial position information in STM interacts with spatial attention: participants were faster to detect a dot located on the right side of a screen if they were concurrently retrieving information from final positions of the memory list; this attentional bias decreased linearly with decreasing recency of the serial position activated in STM. These data indicate that spatial attention processes may also support serial order coding especially in demanding tasks such as STM tasks. The present study may actually provide evidence for both types of processes. The anterior IPS was modulated as a function of ordinal distance across all tasks investigated here and was also found to be active during the encoding-maintenance phases of the order STM task, in line with the intervention of a common ordinal representational system. The posterior IPS, on the other hand, also reacted in a distance sensitive manner, but this only for the more demanding task, i.e., the order STM task. As already noted, the posterior IPS has been associated with attentional control processes during STM tasks, and with the dorsal attention network more generally [27], [28], [44]. This intervention of distance-sensitive attentional processes is in line with the attentional account of serial order proposed by van Dijck and colleagues [59], [60]. Hence, in demanding tasks, both ordinal representational systems supported by the anterior IPS and controlled spatial attention mechanisms supported by the posterior IPS may intervene to encode and process ordinal information.

Finally, as already discussed, distance effects in the IPS could also be the reflection of other processes such as magnitude processing at least for the numerical judgment task. The possibility that the distance effects were not perfectly reflecting the same processes in each task is supported by two observations. First, the behavioral distance effects correlated for the order STM and alphabetical control conditions (and this even after controlling for distance effects in the luminance control condition), but not between these tasks and the numerical processing condition. Also, null conjunction between task-related effects revealed no common activations suggesting that overall different neural networks, and by extension, cognitive processes supported task performance across the three tasks. This raises further questions about the processes that actually drive the commonality of distance-sensitive activations in the IPS across the three tasks. Besides the recruitment of ordinal codes and comparison processes, an alternative hypothesis has been proposed by Franklin and Jonides [61]. These authors observed common IPS activations for ordinal and magnitude numerical processes and suggested that the common IPS activation would reflect the activation of a same “mental number line” used for both, ordinal and magnitude numerical judgment [see also 62]. Although this hypothesis could be plausible for common distance-sensitive activations in the bilateral IPS for the numerical order and the order STM task in the present study, where participants may have represented item positions in the STM list by a numerical equivalent (“this item was in position 1,this item was in position 2, …”), this is much less likely for the alphabetical judgment task; except for perhaps the very first letters of the alphabet, it is unlikely that participants activated numerical codes when comparing the alphabetic order of letters (e.g., for the pair, f–i, it is very improbable that participants activated the information that f and i are respectively, the 6^th^ and 9^th^ letter of the alphabet). Hence, at least as regards common distance-sensitivity of the left IPS across the three tasks considered here, a more general, cross-domain process must be involved. The most plausible interpretation is that this process is related to activation and comparison of ordinal codes, since ordinal processing is the common denominator among the processes potentially involved during the order STM, alphabetical judgment and numerical judgment tasks. A final alternative interpretation is that common distance-sensitivity in the left IPS reflects more general attentional control processes during stimulus comparison, with higher attentional demands for closer and more difficult to distinguish positions, although, as already mentioned, we tried to control for this possibility as much as possible by including the luminance control condition. Furthermore, attentional control is typically associated with more posterior activations than the common distance-sensitive anterior left IPS activations observed here, as already discussed earlier.

To conclude, the present study provides the first compelling evidence for an overlap of neural substrates involved in ordinal coding for STM, alphabetical and numerical domains, suggesting the left aIPS supports common purpose ordinal comparison processes. These findings open new perspectives for the understanding of serial order representation in STM but also across domains.
